# The Gene Expression Profile in the Synovium as a Predictor of the Clinical Response to Infliximab Treatment in Rheumatoid Arthritis

**DOI:** 10.1371/journal.pone.0011310

**Published:** 2010-06-25

**Authors:** Johan Lindberg, Carla A. Wijbrandts, Lisa G. van Baarsen, Gustavo Nader, Lars Klareskog, Anca Catrina, Rogier Thurlings, Margriet Vervoordeldonk, Joakim Lundeberg, Paul P. Tak

**Affiliations:** 1 Department of Gene Technology, School of Biotechnology, AlbaNova University Center, Royal Institute of Technology, Stockholm, Sweden; 2 Division of Clinical Immunology and Rheumatology, Academic Medical Center/University of Amsterdam, Amsterdam, The Netherlands; 3 Rheumatology Unit, Department of Medicine, Karolinska Institute, Karolinska University Hospital, Solna, Sweden; Johns Hopkins School of Medicine, United States of America

## Abstract

**Background:**

Although the use of TNF inhibitors has fundamentally changed the way rheumatoid arthritis (RA) is treated, not all patients respond well. It is desirable to facilitate the identification of responding and non-responding patients prior to treatment, not only to avoid unnecessary treatment but also for financial reasons. In this work we have investigated the transcriptional profile of synovial tissue sampled from RA patients before anti-TNF treatment with the aim to identify biomarkers predictive of response.

**Methodology/Principal Findings:**

Synovial tissue samples were obtained by arthroscopy from 62 RA patients before the initiation of infliximab treatment. RNA was extracted and gene expression profiling was performed using an in-house spotted long oligonucleotide array covering 17972 unique genes. Tissue sections were also analyzed by immunohistochemistry to evaluate cell infiltrates. Response to infliximab treatment was assessed according to the EULAR response criteria. The presence of lymphocyte aggregates dominated the expression profiles and a significant overrepresentation of lymphocyte aggregates in good responding patients confounded the analyses. A statistical model was set up to control for the effect of aggregates, but no differences could be identified between responders and non-responders. Subsequently, the patients were split into lymphocyte aggregate positive- and negative patients. No statistically significant differences could be identified except for 38 transcripts associated with differences between good- and non-responders in aggregate positive patients. A profile was identified in these genes that indicated a higher level of metabolism in good responding patients, which indirectly can be connected to increased inflammation.

**Conclusions/Significance:**

It is pivotal to account for the presence of lymphoid aggregates when studying gene expression patterns in rheumatoid synovial tissue. In spite of our original hypothesis, the data do not support the notion that microarray analysis of whole synovial biopsy specimens can be used in the context of personalized medicine to identify non-responders to anti-TNF therapy before the initiation of treatment.

## Introduction

Rheumatoid arthritis (RA) is a chronic inflammatory disease affecting the synovial tissue in multiple joints. RA is characterized by an influx of inflammatory cells, which leads to hyperplasia and eventually destruction of cartilage and bone [1]. RA is a heterogeneous disease with differences in both disease progression and genetic background of individual patients [2], [3]. The advent of TNF antagonists has revolutionized the treatment of RA, although not all patients respond well [4]. Identification of non-responders is important, not only because anti-TNF treatment elevates the risk for adverse events such as infections [5], but also for financial reasons. Therefore, we previously hypothesized that synovial tissue analysis might be used to predict the response to anti-TNF therapy. Several different approaches have been undertaken in order to predict response to anti-TNF treatment in RA patients, but low success rate leaves room for improvements [6], [7], [8], [9], [10], [11], [12]. Three studies have performed expression analysis with microarrays using RNA extracted from peripheral blood monocytes (PBMCs) with the purpose of predicting response [13], [14], [15]. Only one could detect significant differences between responders and non-responders before treatment [14]. Although the results appeared promising only one of the 20 transcripts was verified by real-time PCR as significant between responders and non-responders at baseline, and previous lack of stability of microarray classifiers [16] warrants verification in an independent study. Previously, microarray technology was also applied on serial synovial biopsies in a study of 10 RA patients to investigate the effects of infliximab treatment on the transcriptional profile with promising results [17]. In another study transcriptional profiling was performed on synovial biopsies obtained at baseline from 18 RA patients before treatment with infliximab [18]. Several biological processes related to inflammation were correlated to a better clinical response. In contrast, another study in 25 RA patients identified a signature of 439 genes mainly associated with cell division and immune response pathways to be associated with the clinical response to adalimumab treatment [19]. Taken together, results of different studies have been variable. Moreover, although differences on the group levels have been suggested, there has been no convincing evidence that this approach could be helpful in predicting clinical response reliably in individual patients. To further investigate whether the molecular signature at baseline could be used to predict the clinical response to anti-TNF therapy in the context of personalized medicine, we performed transcriptional profiling of whole synovial biopsies obtained from 62 RA patients before initiation of infliximab therapy.

## Methods

### Ethics statement

The Medical Ethics Committee of the Academic Medical Center, University of Amsterdam approved the protocol. All patients gave written informed consent.

### Patients

The patients participated in a previously described prospective study [10]; we selected patients from the original cohort based on the presence of sufficient synovial tissue to allow microarray analysis. All patients fulfilled the ACR (American College of Rheumatology) criteria for RA [20], had failed at least two disease-modifying antirheumatic drugs (DMARDs) including methotrexate (MTX), and had a disease activity score evaluated in 28 joints (DAS28) ≥3.2 when included in the study. Patients were on stable maximal tolerable MTX treatment (5–30 mg/week). The use of non-steroidal anti-inflammatory drugs (NSAIDs) and oral corticosteroids (≤10 mg/day) was allowed if stable for at least one month prior to baseline. Concomitant medication was kept stable throughout the study. Previous use of a TNF blocking agent was an exclusion criterion. Baseline characteristics of the patients are given in table 1.

**Table 1:** Baseline patient characteristics.

	All patients	Responders*	Non-responders*	P-value
	(n = 62)	(n = 48)	(n = 14)	
**Demographics**				
Age (years)	55±12	56±13	54±12	0,55
Female (%)	48 (77)	37 (77)	11 (79)	0,91
**Disease status**				
Disease duration (months)	132±114	137±122	116±87	0,55
Erosive disease (%)	45 (73)	37 (77)	8 (57)	0,14
Rheumatoid factor positive (%)	44 (71)	36 (75)	8 (57)	0,2
Anti-CCP positive (%)	46 (74)	38 (79)	8 (57)	0,1
DAS28	6,0±0,9	5,9±0,9	6,2±1,0	0,26
Patients global score (0–100mm)	64±21	61±22	72±16	0,05
ESR (mm/hr)	29±18	31±17	23±19	0,17
C-reactive protein (mg/dL)	16±16	17±16	14±18	0,64
**Drug treatments**				
Previous DMARDs	2,3±1,4	2,2±1,3	2,7±1,7	0,19
Methotrexate (mg/wk)	17,7±8,8	17,2±8,8	19,3±8,7	0,44
Receiving corticosteroids (%)	16 (26)	14 (29)	2 (14)	0,67
Receiving NSAIDs (%)	29 (47)	22 (46)	7 (50)	0,78

*Based on Eular response criteria.

### Treatment and evaluation of clinical response

Patients were treated with infliximab, a commercial antibody marketed by Schering-Plough. There are no patents, marketed products, or products in development related to this research. Also, the use of infliximab does not alter the adherence of this work to all the PLoS ONE policies on sharing data and materials. Infliximab was given in a dosage of 3 mg/kg intravenously at baseline, week 2, 6, and subsequently every 8 weeks. The DAS28 was assessed at baseline, week 4, 8, 12, and 16 by skilled research nurses. Clinical response was evaluated according to the EULAR (European League Against Rheumatism) response criteria [21] at week 16 after initiation of infliximab treatment.

### Synovial biopsy and RNA extraction

**Figure 1:**
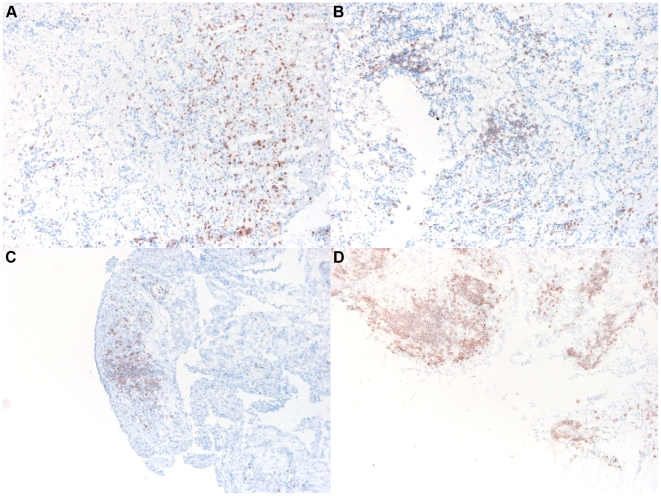
Lymphocyte aggregates. **T**he presence of lymphocyte aggregates was assessed on anti-CD3-stained sections. Aggregates were counted and graded by size. Aggregate size was assessed by counting the number of cells in a radius starting from an estimated center of the aggregate. Aggregate size was then classified as grade 1 (1–5 cells in the radius [panel B]), grade 2 (5–10 cells in the radius [panel C]), or grade 3 (>10 cells in the radius [panel D]). Tissue sections with no lymphocyte aggregates were graded as 0 (panel A).

All patients underwent synovial tissue sampling at the Academical Medical Center/University of Amsterdam by needle arthroscopy of an actively inflamed joint (knee, ankle or wrist), performed under local anesthesia, as described previously in detail [22]. Biopsy specimens were obtained with a 2-mm grasping forceps (Storz, Tuttlingen, Germany) from 6 or more sites within the joint to minimize sampling error. The biopsy specimens (15–50 mg) were snap frozen in liquid nitrogen and homogenised in 1 ml RNA-STAT 60TM reagent (Tel-Test, Friendswood, TX). The quality was assured by measuring the OD260/280 ratio using a Thermo Scientific NanoDrop 1000 spectrophotometer (Thermo Scientific, Wilmington, DE). Samples with a ratio >1.8 were used. A separate block of at least 6 synovial tissue samples from the same patients was snap frozen and assessed for the presence of lymphocyte aggregates, as described previously in detail [23]. Briefly, aggregate size was assessed by counting the number of cells in a radius starting from an estimated center of the aggregate (figure 1). Aggregate size was then classified as grade 1 (1–5 cells in the radius), grade 2 (5–10 cells in the radius), or grade 3 (>10 cells in the radius). Tissue sections with no lymphocyte aggregates were graded as 0. Grade 2 and grade 3 aggregates were termed large lymphocyte aggregates, while grade 1 aggregates were termed small lymphocyte aggregates.

### RNA reference

Universal Human Reference RNA (Stratagene, Cedar Creek, TX) was used as reference RNA. The reference RNA was amplified and labeled according to the same procedure as with the patient material.

### RNA amplification

The RNA was amplified with the RiboAmp II kit (Applied Biosystems, Foster City, CA). 500 ng of total RNA was used as input for the amplification for all except 7 patients. For these patients 300–500 ng was used due to low yield in the RNA extraction step. RNA from 18 good responding, 30 moderate responding and 14 non-responding patients was successfully amplified and subsequently labeled for hybridization.

### Labeling and cDNA synthesis

Labeling was performed with the ULS aRNA Fluorescent Labeling Kit (Kreatech Diagnostics, the Netherlands) that allows for labeling of unmodified aRNA. 2 ug was used as input in the labeling reaction.

### Oligonucleotide microarray

The microarray used in this work is an in-house printed array manufactured at the KTH core facility [24]. The spotted 70-mer oligo nucleotides originate from version 3.03 of Operons Human Genome Oligo Set (Operon, Huntsville, AL). The microarray contains 35344 features (spots) representing 28948 Entrez Gene IDs [25] of which 17972 are unique.

### Hybridization

Pre-hybridization of microarray slides was previously described [24]. Fragmentation was performed after labeling of the aRNA with RNA fragmentation reagents AM870 (Applied Biosystems) yielding aRNA fragment sizes of 60–200 nucleotides. The cy5 and cy3 labeled aRNA were then pooled in equivalent amounts and mixed with the hybridization buffer consisting of 5× SSC, 25% formamide (Sigma-Aldrich, St. Louis, MO) 0.1% SDS, 25% Kreablock (Kreatech Diagnostics), 10 ug herring sperm DNA (Invitrogen, Carlsbad, CA) and 10 ug yeast tRNA (Invitrogen). The hybridization mixture was then heat denatured in 70°C for 3 minutes and subsequently put on ice until injection in a MAUI hybridization mixer (Biomicro, Salt Lake City, UT). The hybridization was performed over night in the MAUI hybridization station (Biomicro) in which the hybridization mixture is stirred to increase sensitivity and specificity. Subsequent washing steps were performed as previously described [26].

### Scanning and image processing

Scanning and image processing was performed as previously described [24]. Briefly, the microarrays were scanned using the Agilent G2565BA scanner (Agilent Technologies, Palo Alto, CA) and the feature intensities were extracted using the GenePix 5.1.0.0 software (Axon Instruments, Sunnyvale, CA).

### Experimental design and data analysis

All patients were hybridized in a reference design where the reference was always labeled with cy3 and the samples with cy5 [27]. The microarray data were analyzed in R, an environment for statistical programming and computing [28]. Several freely available R packages were used for low-level analysis and normalization [29] as described previously [24], [26]. SAM (Significance Analysis of Microarrays), available as the samr package in R, was applied to identify differentially expressed (DE) genes [30]. Since SAM does not allow for blocking, the LIMMA (Linear Models for Microarray Data) [31] package, present in Bioconductor [29], was applied to control for the effect of ectopic lymphocyte aggregates when comparing responders. Hierarchical clusters were performed using 1 – the Pearson correlation as the distance measure [32]. Enrichment analysis was carried out to identify biological themes in groups of relevant genes [33] using the the Gene Ontology and KEGG databases [34], [35]. PAGE (Parametric Analysis of Gene set Enrichment) was also used to test for significant pathways/categories between response groups in the above-mentioned databases [36], [37]. FDR was used to correct for multiple testing in all enrichment/pathway analyses [38]. In all tests using the FDR, a q-value (the FDR analog of a p-value) <0.05 was used as a cut-off for statistical significance. A two-sided binomial exact test was used to test for overrepresentation of lymphoid aggregates within response groups assuming a 0.5 chance of having an aggregate or not. The microarray data set has been deposited at Gene Expression Omnibus [39] under accession number GSE21537.

## Results

### Significance analysis of microarrays (SAM)

SAM [30] was used to query differences between response groups prior to treatment. The followings groups were defined according to the EULAR response criteria: good responders (G, n = 18), moderate responders (M, n = 30), responders (G and M together: R, n = 48), and non-responders (N, n = 14). No features were differentially expressed (DE, q-value<0.05) between the different response groups (G vs. N, G vs. M, M vs. N, R vs. N). Although individual features did not reach statistical significance between response groups, PAGE [36] disclosed GO categories (e.g. GO:0006935, chemotaxis, q-value 5e-50; GO:0006954 inflammatory response, q-value 2e-46; GO:0045321, leukocyte activation, q-value 3e-23) with a higher average level in G vs. N, which indicates a general higher level of active inflammation in G. In addition, patients were split into two cohorts based on the presence of anti-citrullinated protein antibodies (ACPA). There were no statistically significant differences based on the EULAR response in either ACPA positive or negative patients. Collectively, at the group level expression levels of genes involved in inflammatory processes are enriched in G, but no predictive biomarkers could be identified that could serve as surrogate markers of response to anti-TNF treatment in individual patients.

### Hierarchical cluster of patients using features with high variation

**Figure 2:**
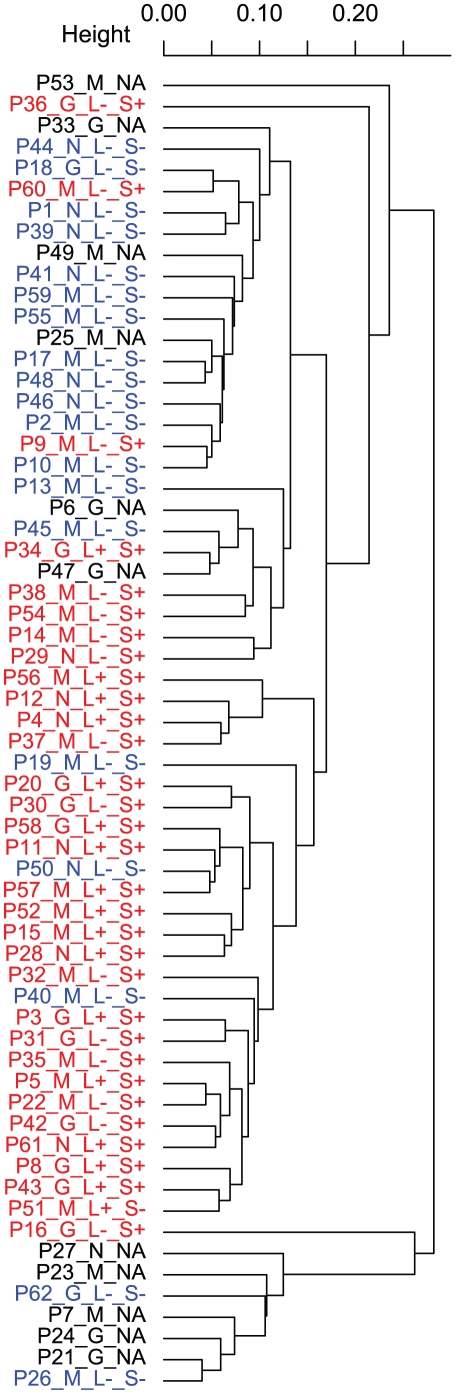
Hierarchical cluster of patients using features with high variation irrespective of response group. Patients were clustered in a hierarchical dendrogram using features with a log_2_-ratio (ratio = sample intensity/reference intensity) interquartile range >1 (1321 features in total). Abbreviations in the dendrogram: PXX = Patient number; G,  = Good responder; M = Moderate responder; N = Non responder; L+/− = presence of large lymphocyte aggregates; S+/−presence of small aggregates. Colors in the dendrograms: Red = patients positive for either small or large lymphocyte aggregates; Blue = patients negative for small and large lymphocyte aggregates; Black = patients with no lymphocyte aggregate assessment.

To get an overview of the variation between samples a hierarchical clustering was performed using features with high variance (log_2_-fold change values with interquartile range >1) independent of response group (1321 features in total, figure 2). The patients did not cluster according to the EULAR response criteria, but the presence of synovial lymphocyte aggregates (both small and large) dominated the pattern of the dendrogram. Enrichment analysis [33] was performed on the 1321 features using the Gene Ontology (GO) and KEGG databases [34], [35] which revealed themes indicative of inflammatory disease (top GO category, GO:0006955, Immune response, q-value 3.2e-11; top KEGG category, 04612, Antigen processing and presentation, q-value 2.3e-06). Thus, the synovial gene expression profile is especially characterized by the level of cell infiltration and immune activation.

### The effect of the presence of lymphocyte aggregates

Lymphocyte aggregate data were available for 51 out of all 62 patients. The detailed description of lymphocyte aggregates in relationship to clinical response to infliximab treatment has been reported before in detail for the larger cohort and we have shown that RA patients with synovial lymphocyte aggregates have on average a better response to infliximab treatment than those with only diffuse leukocyte infiltration [40]. In the 51 patients included in the present study lymphocyte aggregates (small and/or large) were detected in 11/13 (85%), 15/25 (60%), and 6/13 (46%) of G, M and, N, respectively. Only G had a significantly higher level of aggregates (small and/or large, binomial exact test; G, p = 0.02; M, p = 0.4; N, p = 1). Therefore, patients with aggregates were tested for statistically significant differences in transcriptional profile compared to those without aggregates, irrespective of response group. Here, 1965 features were significantly different between the two groups (q-value<0.05). Enrichment analysis of the top 500 DE genes revealed relevant and significant themes (Table 2). Since response was positively correlated to the presence of aggregates (small and/or large, Pearson correlation, r = 0.3 (p-value: 0.04) and there were significant differences between aggregate positive and negative patients, a linear model was set up in LIMMA [31] to control for the effect of small and/or large aggregates when comparing response groups responders (G vs. N, G vs. M, M vs. N, R vs. N), after which we did not detect any statistically significant differences. Next, we stratified the patients based on the presence of lymphocyte aggregates (small and/or large aggregates versus no aggregates) and examined the relationship between transciptional profile and clinical response to infliximab for each tissue group. The division into aggregate positive versus negative patients markedly decreased the sample sizes; therefore, we could only compare R to N. In all comparisons no features were found to be DE except for 38 features comparing G versus N in the aggregate positive group (Table S1). Enrichment analysis revealed no significant KEGG pathways, but two GO categories were significant (GO:0006412, translation, q-value 0.02; GO:0009058, biosynthetic process, q-value 0.02).

**Table 2:** Enrichment analysis between patients with aggregates or not.

KEGG			
KEGG ID	q-value	Count	Term
4060	0,042	15	Cytokine-cytokine receptor interaction
4660	0,042	9	T cell receptor signaling pathway
5216	0,042	5	Thyroid cancer
4650	0,042	10	Natural killer cell mediated cytotoxicity

Gene Ontology			
GO ID	q-value	Count	Term
GO:0006955	3,40E-12	38	immune response
GO:0002521	5,70E-06	13	leukocyte differentiation
GO:0001775	2,10E-03	15	cell activation
GO:0048534	1,20E-02	13	hemopoietic or lymphocyte organ development
GO:0009607	1,70E-02	15	response to biotic stimulus
GO:0045619	1,70E-02	5	regulation of lymphocyte differentiation
GO:0051239	4,90E-02	16	regulation of multicellular organismal process

KEGG ID, GO ID, ID for the KEGG/Gene Ontology databases [34], [35]; q-value, the false discovery rate; Count, the number of genes mapping to the specific term/pathway among the 500 tested; Term, the name in the respective database.

## Discussion

The efficiency of TNF blockade might theoretically be increased if patients can be identified who will not respond to TNF blocking agents, before the initiation of anti-TNF therapy. In the ∼30% patient who would not receive anti-TNF therapy on the basis of a biomarker-directed strategy, unnecessary and expensive parenteral therapy could be withheld. In addition, therapies with a different mechanism of action, like rituximab, abatacept, or tocilizumab could be initiated in these patients earlier in the disease course than according to the current treatment algorithm. The combination of multiple markers bears most promise to improve the performance of a biomarker-guided approach, as it could reduce the extensive overlap in individual marker levels that exists between responders and non-responders. Conceivably, analysis of gene expression profiles could provide a molecular signature that may be used to predict the response to treatment, similar to its use to predict outcome in patients with breast cancer [41]. The study described here was performed in 62 RA patients, which is a marked increase in scale from previous studies using synovial biopsies to investigate gene expression differences between different response groups to anti-TNF treatment. The question was if the larger number of patients would allow for the identification of possible biomarkers that could be used in individual patients. Of importance and in contrast to general belief, this does not seem to be the case. Moreover, this study identified an important confounding factor that is relevant for studies using microarray analysis of RA synovial tissue: the presence of lymphocyte aggregates. When clustering the patients to get an overview of the variation between responders, the presence of ectopic aggregates was the most discriminating variable. Since the presence of aggregates correlated with response, all comparisons with regard to transcriptional profile between good, moderate and non-responders were confounded. Recent studies have suggested that lymphocyte aggregates are not necessarily functioning as canonical germinal centers, but may occur as a secondary effect to chronic inflammation [23], [42]. The overrepresentation of aggregates in good responders is in line with the earlier described relationship between synovial inflammation and the response to infliximab treatment [10]. Considering that aggregates were positively correlated to response and since the largest detectable difference was between aggregates, we set up a linear model to control for the effect of aggregates. Again, we could not detect any significant differences. This could indicate that different pathogenic pathways are active in aggregate positive versus negative patients. Therefore, the patients were split into aggregate positive and negative patients. Here 38 features were statistically significant between good and non-responding patients in the aggregate positive group. The enrichment analysis is not easily explained, containing many ribosomal transcripts (the GO categories translation and biosynthetic process were significantly enriched). This could argue for a higher metabolism rate in good responding patients, indicative of a higher level of inflammation, as shown in our previous work. Taken together, the results presented here clearly show that in microarray analysis studies of whole RA synovium, it is critical to control for the presence of lymphocytes aggregates, as they may represent an important confounding factor. Moreover, the results do not support the notion that microarray analysis of whole synovial biopsies may be used to predict the response to anti-TNF treatment in the context of individualized medicine. Our findings are also in agreement with the clinical experience that the response to TNF blockade is not a dichotomous phenomenon [43]. In fact, even so-called non-responders may exhibit protection against joint destruction [44], illustrating that clinical responders and non-responders to anti-TNF therapy do not necessarily represent completely distinct pathogenetic subsets of RA. We need to consider that transcription analysis of synovial biopsies results in an expression profile that originates from a mixture of cells. Future work should address the question as to whether analysis of individual cell types derived from the synovium might yield better predictive biomarkers. Previous work, using immunohistochemistry, has demonstrated a correlation between clinical response to anti-TNF treatment on the one hand and TNF protein expression as well as expression of cells capable of producting TNF on the other. [10]. Although the correlation of TNF on the protein level does not automatically entail the same distinct transcriptional patterns due to regulation [45], [46], it is conceivable that the expression profile of microdissected cell populations, previously known to be correlated with response, may provide better targets for finding predictive biomarkers.

## Supporting Information

Table S1 This table contains the 38 features that were differentially expressed comparing good- and non-responding patients in the aggregate positive group. The table headings are: chip ID - the unique identifier on the microarray; q-value in percent - the false discovery rate; log2 fold difference - the fold change of gene expression between good vs. non-responding patients on a log-2 scale; Entrez gene ID - an identifier from the Entrez gene database [1]; Gene Name - an identifier from the the Human Gene Nomenclature Database [2]. NA - not available, displayed for features with no annotation for the column in question.
(0.07 MB DOC)
