# Supplementary material for: The Gene Expression Profile in the Synovium as a Predictor of the Clinical Response to Infliximab Treatment in Rheumatoid Arthritis
Source: PLoS One. 2010 Jun 25;5(6):e11310. doi: 10.1371/journal.pone.0011310 (PMC2892481; doi:10.1371/journal.pone.0011310)
Supplement: Table S1 — This table contains the 38 features that were differentially expressed comparing good- and non-responding patients in the aggregate positive group. The table headings are: chip ID - the unique identifier on the microarray; q-value in percent - the false discovery rate; log2 fold difference - the fold change of gene expression between good vs. non-responding patients on a log-2 scale; Entrez gene ID - an identifier from the Entrez gene database [1]; Gene Name - an identifier from the the Human Gene Nomenclature Database [2]. NA - not available, displayed for features with no annotation for the column in question. (0.07 MB DOC) [file pone.0011310.s001.doc]

| Chip ID | qvalue (percent) | log2 fold difference | Entrez Gene ID | Gene Name |
| --- | --- | --- | --- | --- |
| Humv312J14 | 0,0 | 1,3 | 3956 | lectin, galactoside-binding, soluble, 1 (galectin 1) |
| Humv363J05 | 0,0 | -0,4 | 6338 | sodium channel, nonvoltage-gated 1, beta (Liddle syndrome) |
| Humv321C18 | 0,0 | -2,3 | 51053 | geminin, DNA replication inhibitor |
| Humv331E08 | 3,1 | 0,6 | 23022 | palladin, cytoskeletal associated protein |
| Humv320F14 | 3,1 | 1,2 | NA | NA |
| Humv353I16 | 3,1 | 1,5 | 51673 | tubulin polymerization-promoting protein family member 3 |
| Humv380G10 | 3,1 | 0,4 | NA | NA |
| Humv312J12 | 3,1 | 0,9 | 3956 | lectin, galactoside-binding, soluble, 1 (galectin 1) |
| Humv345F18 | 3,1 | 0,6 | 4841 | non-POU domain containing, octamer-binding |
| Humv363E21 | 3,1 | 0,6 | 10476 | ATP synthase, H+ transporting, mitochondrial F0 complex, subunit d |
| Humv332G11 | 3,1 | 0,4 | 25796 | 6-phosphogluconolactonase |
| Humv301B07 | 3,1 | 0,5 | 7311 | ubiquitin A-52 residue ribosomal protein fusion product 1 |
| Humv368I12 | 3,1 | 0,9 | 6206 | ribosomal protein S12 |
| Humv374I11 | 3,1 | 0,9 | 220717 | ribosomal protein P0-like |
| Humv341H20 | 4,3 | 0,4 | 51529 | APC11 anaphase promoting complex subunit 11 homolog (yeast) |
| Humv388B15 | 4,3 | 1,6 | 643834 | pepsinogen 3, group I (pepsinogen A) |
| Humv316P01 | 4,3 | 0,4 | 51398 | chromosome 19 open reading frame 56 |
| Humv388G12 | 4,3 | 0,5 | NA | NA |
| Humv311M01 | 4,3 | 0,5 | 51168 | myosin XVA |
| Humv334N06 | 4,9 | 1,2 | NA | NA |
| Humv357K18 | 4,9 | 0,4 | 9553 | mitochondrial ribosomal protein L33 |
| Humv392K01 | 4,9 | 1,4 | NA | NA |
| Humv388O06 | 4,9 | 0,4 | 646208 | similar to hCG1782986 |
| Humv377K06 | 4,9 | 0,5 | 2303 | forkhead box C2 (MFH-1, mesenchyme forkhead 1) |
| Humv327B08 | 4,9 | 0,6 | NA | NA |
| Humv316O22 | 4,9 | 0,6 | 28974 | chromosome 19 open reading frame 53 |
| Humv356M23 | 4,9 | 0,5 | 3020 | H3 histone, family 3A |
| Humv395J04 | 4,9 | 0,8 | 2191 | fibroblast activation protein, alpha |
| Humv395I10 | 4,9 | 0,5 | 10758 | TRAF3 interacting protein 2 |
| Humv303M18 | 4,9 | 0,5 | 56895 | 1-acylglycerol-3-phosphate O-acyltransferase 4 (lysophosphatidic acid acyltransferase, delta) |
| Humv355N19 | 4,9 | 0,4 | NA | NA |
| Humv314H21 | 4,9 | 1,1 | 6173 | ribosomal protein L36a |
| Humv333P01 | 4,9 | 1,0 | 54453 | Ras and Rab interactor 2 |
| Humv346F22 | 4,9 | 0,7 | 23521 | ribosomal protein L13a |
| Humv337C01 | 4,9 | 0,4 | 341676 | NIMA (never in mitosis gene a)-related kinase 5 |
| Humv387D21 | 4,9 | 0,3 | NA | NA |
| Humv345J15 | 4,9 | 0,4 | 6129 | ribosomal protein L7 |
| Humv375L07 | 4,9 | 0,5 | NA | NA |

**Supplementary table 1.**

This table contain the 38 features that were differentially expressed comparing good- and non-responding patients in the aggregate positive group. The table headings are: chip ID – the unique identifier on the microarray; q-value in percent – the false discovery rate; log2 fold difference – the fold change of gene expression between good vs. non-responding patients on a log-2 scale; Entrez gene ID – an identifier from the Entrez gene database [1]; Gene Name – an identifier from the the Human Gene Nomenclature Database [2]. NA – not available, displayed for features with no annotation for the column in question.

1. Maglott D, Ostell J, Pruitt KD, Tatusova T (2005) Entrez Gene: gene-centered information at NCBI. Nucleic Acids Res 33: D54 58.

2. Wain HM, Lush M, Ducluzeau F, Povey S (2002) Genew: the human gene nomenclature database. Nucleic Acids Res 30: 169-171.
